# Beyond single-person paradigms: N400 alterations in a social context in schizophrenia

**DOI:** 10.3389/fpsyt.2026.1922640

**Published:** 2026-09-04

**Authors:** Máté Fullajtár, Brigitta Kakuszi, István Bitter, Pál Czobor

**Affiliations:** Department of Psychiatry and Psychotherapy, Semmelweis University, Budapest, Hungary

**Keywords:** EEG, hyperscanning, N400, schizophrenia, social cognition

## Abstract

**Introduction:**

Impairments in social cognition and context-dependent information processing constitute prominent features of schizophrenia. Among event-related potentials (ERPs), the N400 is known to be sensitive to semantic and social contextual information. However, its role in social contexts has received limited attention in schizophrenia research, as previous studies have largely examined the N400 in isolated, single-person paradigms or have focused on the P300 component.

**Methods:**

In this study, we examined the N400 ERP component in a real-world social context using EEG hyperscanning. Patients with schizophrenia (SZ; N = 34) and healthy controls (HC; N = 48) performed an actor–observer Go/NoGo task in pairs using the International Affective Picture System (IAPS) with emotionally valenced and neutral images. The task was repeated, with participants switching roles, and analyses were restricted to the actor condition at fronto-central electrodes. Clinical symptoms were assessed using the Positive and Negative Syndrome Scale (PANSS). Although our analysis focused on the N400, we also examined the P300 amplitude, given the functional relationship between these components during information processing.

**Results:**

Primary analysis revealed significantly reduced N400 amplitudes in patients with schizophrenia compared with healthy controls, both across all stimuli and specifically in response to negative and positive stimuli. With second stimulus exposure, N400 amplitudes remained stable in healthy controls but showed a significant decline in the schizophrenia group. During the second exposure, higher PANSS total scores and higher scores on the negative and general psychopathology subscales were significantly associated with attenuated N400 amplitudes, whereas lower levels of psychopathology were associated with a polarity reversal. Secondary analyses revealed significantly reduced P300 amplitudes in patients with schizophrenia for positive stimuli and for all stimulus-valences combined. With second exposure, P300 amplitudes increased in the schizophrenia group, whereas they decreased in healthy controls.

**Discussion:**

The observed alterations in N400 responses indicate that impairments in context-dependent affective information processing in schizophrenia extend to social context. Alterations in the P300 component may further reflect deficits in cognitive control and response inhibition. Collectively, these findings suggest a dysfunction in neural processes responsible for monitoring and resolving mismatches between internal predictions and social contexts.

## 1 Introduction

Schizophrenia is a chronic psychiatric disorder characterized by a cluster of symptoms. Based on clinical presentation, we can distinguish between positive (e.g., delusions), negative (e.g., emotional blunting), and cognitive symptoms. Impaired social cognition has also been described in the literature in patients with schizophrenia (1, 2). Social cognition is the process and function that enables the appropriate interpretation of interpersonal interactions. It is an important determinant of the functional outcome of the illness, as it plays a mediating role between neurocognitive functions and everyday functionality and social functioning (3–5).

Patients with schizophrenia frequently exhibit impairments in social cognition and context-dependent information processing. Among event-related potentials (ERPs), the N400 component is particularly sensitive to these processes (6–8). The N400 wave is a negative-amplitude deviation that occurs in the time window between 200 and 600 milliseconds following the onset of the stimulus, reaching its peak at approximately 400 milliseconds. The N400 typically appears in response to contextually incongruent stimuli; according to more recent theories, it is a neural marker of prediction error (9–11).

While the N400 ERP component was originally described in linguistic paradigms, it can also be observed in response to stimuli presented in visual or social contexts (6). Since previous studies have also shown that N400 effects associated with action semantics and social-contextual integration may exhibit frontal or fronto-central scalp distributions, this suggests the involvement of higher-order predictive and integrative processes (6).

As an electrophysiological index of semantic priming—which has also been shown to be modulated during action observation and comprehension (6)—the N400 reflects semantic network activation and the strength of connections between contextual elements. Its amplitude is greatest (i.e., most negative) for unrelated information, decreases for related stimuli, and nearly disappears during the processing of semantically matching information. Hyperpriming refers to a diffuse activation of the semantic network, wherein semantic nodes respond not only to closely associated stimuli but also to loosely associated or even unrelated stimuli. A characteristic feature of this phenomenon is that the amplitude of the N400 is reduced in the presence of inconsistent stimuli. From a clinical perspective, this can be linked to characteristic symptoms of psychosis, such as impaired associative processing, thought disorders, and delusions (10, 11). Although evidence suggests that patients with schizophrenia exhibit a reduced N400 amplitude, there is mixed data regarding the relationship between N400 amplitude abnormalities and psychopathological symptoms; some studies have found differences related to positive or negative symptoms, while others have not found similar associations (12–14). Notably, these N400 abnormalities are not limited exclusively to schizophrenia, as they are also characteristic of other conditions involving psychotic symptoms, including mood disorders and substance use disorders (10).

In addition to the N400, numerous ERP studies have demonstrated an association between the P300 component and symptoms of schizophrenia, findings that have also been summarized in several meta-analyses (15, 16). Among the potentially relevant factors, the relationship between P300 amplitude abnormalities and positive symptoms may be associated with cognitive impairment and sensory gating dysfunction. According to the sensory gating theory, the dysfunction of sensory filters results in information overload in patients with schizophrenia, leading to abnormal experiences that manifest as secondary consequences in clinical settings (17). Additionally, the phenomenon of aberrant salience can also lead to erroneous information processing. According to recent formulations of salience theory, increased subcortical dopamine transmission can induce maladaptive changes, significantly altering the perception of reality. This can lead to abnormal attribution of meaning, pathological associations (thought processes), and the development of delusions and hallucinations. Collectively, these processes may contribute to the intensification of hyperpriming (18, 19). Recent accounts suggest that phenomena such as hypopriming and hyperpriming may reflect fundamental disturbances in the neural mechanisms underlying context-based prediction and the integration of incoming information (19).

This predictive and integrative failure is particularly critical given that patients with schizophrenia often have difficulty recognizing and interpreting emotions, particularly negative ones such as fear, anger, or sadness. This impairment is a fundamental feature of the social cognitive deficits associated with illness, significantly contributing to disturbances in social functioning and interpersonal relationships (20, 21). However, although social settings are a central part of everyday behavior, most schizophrenia research has traditionally relied on isolated, single-person task settings (22–24). Consequently, these single-subject designs cannot shed light on whether the alterations observed in isolation are also observable in social contexts.

Hyperscanning offers a novel approach to investigating the neurobiological basis of social interaction (25, 26). By analyzing the neural activity of two or more individuals within a shared social context, this method serves as a crucial bridge between psychiatry and cognitive neuroscience (25, 27). While various imaging modalities—such as functional magnetic resonance imaging (fMRI), magnetoencephalography (MEG), and near-infrared spectroscopy (NIRS) — are used in hyperscanning designs, electroencephalography (EEG) offers excellent temporal resolution and high ecological validity, enabling the investigation of event-related potentials under real-world conditions (25, 28, 29).

To examine whether predictive abnormalities extend beyond traditional single-person paradigms, we investigated ERP responses in a social context. Although our primary focus was the N400, we also examined the P300 component, as growing evidence indicates that these ERPs are supported by partially overlapping neural processes and jointly contribute to the detection and evaluation of contextual incongruence (30).

To address these gaps in the literature, our aim was to characterize the neurobiological basis of N400 and P300 abnormalities in schizophrenia within a social setting, specifically during responses to visual emotional stimuli. In our study, we employed an EEG-based hyperscanning setup using a Go/NoGo task within an observer-actor paradigm, a framework that allows for the investigation of deficits in behavioral response inhibition and impairments in executive functions in social contexts (31).

## 2 Materials and methods

### 2.1 Participants

A total of 88 participants aged 18–65 were recruited for this exploratory study: 36 inpatients diagnosed with schizophrenia (SZ, 18 pairs) at the Department of Psychiatry and Psychotherapy at Semmelweis University, and 52 healthy control subjects (HC, 26 pairs). The data of two patients and four controls were excluded from subsequent analyses due to signal artifacts. Accordingly, the final sample consisted of 27 men (79%) and 7 women (21%) in the patient group, and 17 men (35%) and 31 women (65%) in the control group. The mean age was 36.4 years (SD = 11.7) for patients and 30.1 years (SD = 9.4) for controls.

The diagnosis of schizophrenia was established based on the DSM-5 criteria (32). In both groups, exclusion criteria included a history of severe neurological or somatic disease (e.g., head injury) and alcohol or drug use prior to the EEG examination. Due to their hospitalization, patients remained abstinent from substances throughout the study period.

Inclusion in the control group required the absence of a psychiatric diagnosis, as well as a score on the 90-item SCL-90R symptom inventory (33) that was below the clinical cutoff value for the general Hungarian population (34). Patients with schizophrenia received medication, the dosage of which was determined during the study in chlorpromazine-equivalent (CPZ) units (35). In accordance with the principles of the Declaration of Helsinki, participants provided written informed consent based on a protocol approved by the local ethics committee (Ethics Committee of Semmelweis University, #116481/AOPSI/2018).

### 2.2 Measurement of psychopathology

The severity of schizophrenic symptoms was measured using the Positive and Negative Syndrome Scale (PANSS). This 30-item questionnaire includes a 7-item positive symptom subscale, a 7-item negative symptom subscale, and a 16-item general psychopathology subscale (36). Each item was scored on a scale of 1 to 7, so the total scores for the positive and negative subscales range from 7 to 49, those for the general psychopathology subscale from 16 to 112, while the total score can range from 30 to 210.

Using a 5-dimensional factor model, the PANSS scale is also suitable for a more detailed characterization of the psychopathological profile. The PANSS is widely evaluated based on a five-factor structure comprising positive symptoms, negative symptoms, disorganized thinking, uncontrolled hostility/agitation, and depression/anxiety (37, 38). The PANSS assessment was conducted in the form of a semi-structured clinical interview by a clinician experienced in using the tool.

### 2.3 Stimulus paradigm

During the study, we examined two participants simultaneously: they sat next to each other in a dimly lit room, in front of separate monitors, approximately 100 cm away from the screen. As a stimulus task, we employed a Go/NoGo paradigm, using images from the International Affective Picture System (IAPS) (39, 40) with negative, positive, or neutral emotional valence. The stimuli were presented using Presentation 13.0 software (Neurobehavioral Systems, Inc., Albany, California, USA).

Participants were instructed to press a key as quickly as possible whenever an image appeared (Go condition), unless the image was identical to the one previously displayed (NoGo condition). The stimulus cycle lasted 1400 ms: each image was visible for 800 ms, followed by a 600-ms inter-stimulus interval. A total of 480 stimuli were presented to each participant, divided into two blocks (240 images per block). The ratio of Go to NoGo tasks was 85% to 15%, respectively.

During the presentation of the stimuli, both participants saw the same images simultaneously, but one served as an observer and the other as an actor. The actors actively performed the task, while the observers watched their own screens as the actors worked. Roles were subsequently reversed to ensure that each participant experienced both conditions, with the order of conditions counterbalanced across pairs (i.e., 50% of participants started as actors during the first exposure, and 50% as observers).

### 2.4 EEG recording and data pre-processing

EEG signals were recorded using two active-electrode amplifier systems (Biosemi Inc., Amsterdam, Netherlands), each with 256 channels, at a sampling rate of 512 Hz. The BioSemi ActiveTwo system is specifically designed to support hyperscanning through optical daisy-chaining of multiple amplifier (AD-box) modules. In our setup, two 128-channel amplifier modules were connected in daisy-chain mode, with one amplifier assigned to each participant. Consequently, each participant due to the daisy chain connection was recorded with 128 EEG channels (41, 42). The amplifier modules are synchronized and their digitized data are transmitted through a single optical interface to the acquisition computer. According to the BioSemi system specifications, this architecture supports the full number of recording channels at the selected sampling rate without data compression or degradation of signal quality, and therefore does not constitute a data-transfer bottleneck. This daisy-chain configuration is part of the standard BioSemi ActiveTwo hyperscanning implementation.

The signal was filtered using a bandpass filter between 0.5 and 100 Hz with an average reference. The electrophysiological data were preprocessed using version 5.1 of the Electro-Magnetic Source Signal Imaging Suite (EMSE Suite) software. During raw data processing, we used an IIR Butterworth filter (0.5–70 Hz) as a bandpass filter, and we eliminated power line noise using a Parks-McClellan notch filter (48–52 Hz). After correcting for artifacts caused by eye movements and filtering out baseline fluctuations using a third-order polynomial filter, the recordings were also manually reviewed offline. ERP analysis was based on stimulus-locked EEG epochs of 1000 ms, comprising a 200 ms pre-stimulus baseline and an 800 ms post-stimulus interval. Epochs containing artifacts exceeding ±100 μV were excluded from further analysis. The remaining epochs were baseline-corrected using the 200 ms pre-stimulus interval and averaged separately for each participant and emotional valence to obtain the ERP waveforms.

Our region of interest (ROI) included electrode Fz and the adjacent electrodes surrounding Fz in the BioSemi recording system, corresponding to the mid-anterior, midline, and lateral electrodes in the International 10–20 System. The voltage values were collapsed (averaged) across these electrodes for the statistical analyses; in the current paper, we refer to this ROI as “fronto-central”.

### 2.5 Statistical analyses

#### 2.5.1 Basic demographic characteristics and behavioral measures

The chi-square test was used to compare gender distributions between the schizophrenia and healthy control groups. General linear model (GLM) analysis was applied to compare the two study groups with respect to age and behavioral data obtained during the actor conditions, including commission and omission errors, and reaction times. A separate analysis was conducted for each of the behavioral measures, used as a dependent variable in the GLM model. Study group (SZ, HC) was used as an independent variable (between-subjects factor). Age, gender and education level were used as covariates in the analyses for the behavioral measures.

#### 2.5.2 ERP measures

Our primary statistical analysis focused on the N400 component. The group difference between schizophrenia and healthy control subjects in the N400 was based on the random regression hierarchical linear mixed-effects model with fixed and random effects (HLM) (43, 44). Model parameters were estimated using restricted maximum likelihood (REML). To account for the non-independence of observations within hyperscanning pairs, the model included a random intercept for dyad (participant pair). In addition, a random intercept for participant nested within dyad accounted for repeated measurements obtained from each participant. The definition of fronto-central N400 component time-window, based on the peak value and the surrounding 100 msec window, included the post-stimulus epoch of 350–450 ms. Amplitude (voltage) values within this time-window of interest were used as dependent variable in the HLM. Study group (SZ, HC) was applied as an independent variable (between-subjects factor) in the model. Time (sampling point in the component window, relative to stimulus onset) was included in the analysis as a within-subject factor. Participant-specific random intercepts accounted for repeated observations; residual temporal autocorrelation was modeled using an autoregressive [AR(1)] covariance structure.

Based on prior literature for N400 in various settings (6), the region of interest included electrode Fz and the adjacent electrodes surrounding Fz in the BioSemi recording system, i.e., the three mid-anterior electrodes in the International 10–20 System, and the midline and lateral electrodes between them (the latter, labeled as C22, C11, and C24, respectively, in the BioSemi-layout). Study group (SZ vs. HC) and exposure (first vs. second exposure to the task) and their interaction were used as the principal independent variables of interest. Exposure was used as an additional within-subject factor to indicate whether the particular ERP response was obtained from the first as opposed to the second exposure to the task. Age and gender served as covariates in the analyses. Correction for multiple testing for the primary analyses which included the full set of group comparisons for the N400 component was performed using the False Discovery Rate (FDR) procedure (45). To facilitate the interpretation of the results, covariate-adjusted Cohen’s *d* effect sizes for the differences between the study groups are provided.

In secondary analyses, we further examined whether differences between the two study groups were also reflected in P300 amplitude. The frontal scalp regions of interest defined for the fronto-central NoGo P300 were identical to those used for the N400 analyses, and the HLM analyses for the P300 followed the same analytical approach as those conducted for the N400. However, the time window of interest for the P300 was earlier (220–320 ms), reflecting the earlier emergence of this component following stimulus onset compared to the N400. All statistical tests were two-sided. The SAS 9.4 version was used for statistical analyses.

For analyses examining the associations between ERP amplitudes and clinical covariates (including psychopathology, demographic characteristics, and medication use), each covariate was entered separately as a fixed effect in the HLM. For analyses examining associations between ERP amplitudes and clinical covariates, Cohen’s *d* effect sizes were derived from the fitted regression model for predefined contrasts in the covariates. For continuous covariates (e.g., PANSS total score), the reported effect size corresponds to a 30-point increase, equivalent to a 1-point increase across all 30 PANSS items. For the PANSS subscales, analogous predefined contrasts were used, corresponding to 1-point increase of all constituting items of the subscale. Positive and negative values of Cohen’s *d* indicate the direction of the association between the covariate and ERP amplitude. For binary covariates (e.g., benzodiazepine use), the effect size represents the standardized difference between the two groups.

## 3 Results

### 3.1 Demographic and clinical data

The descriptive statistical data for the study participants are summarized in **Table 1**. The mean age differed significantly between groups, with patients with schizophrenia being older than healthy controls (SZ: 36.4 years, SD = 11.7; HC: 30.1 years, SD = 9.4). A significant group difference was observed in gender distribution and educational attainment; the SZ group showed a higher proportion of male participants, whereas the HC group exhibited a more balanced gender distribution. Regarding education, a lower proportion of participants with higher educational attainment was observed in the SZ group compared to controls. At the behavioral level, patients with schizophrenia showed significantly longer reaction times in the Go/NoGo task compared to healthy controls (SZ: 614.4 ms, SD = 105.9; HC: 423.8 ms, SD = 62.7). In addition, the SZ group demonstrated significantly higher omission and commission error rates relative to the HC group (omission errors: SZ: 20.4%, SD = 21.7% vs. HC: 1.1%, SD = 2.0%; commission errors: SZ: 32.2%, SD = 17.6% vs. HC: 9.8%, SD = 11.2%).

**Table 1:** Basic demographic and clinical characteristics of the study sample^a^.

Characteristics	HC (N = 48)b	SZ (N = 34)c	Statistic	p
Categorical variables: N (%) [Test: Chi2]				
Demographic: Male/Female, N (%)	17/31 (35.4/64.6)	27/7 (79.4/20.6)	15.49	<.001
Education level (1/2/3)^d^	0/34/14 (0/70.83/29.17)	13/19/2 (38.24/55.88/5.88)	24.57	<.001
Continuous variables: Mean (SD) [Test: F]				
Age (years)	30.1 (9.4)	36.4 (11.7)	7.31	.008
PANSS total score	-n/a	101.6 (20.7)	-n/a	–
Positive subscale score (sum of 7 items)	–	23.7 (6.3)	–	–
Negative subscale score (sum of 7 items)	–	25.3 (5.9)	–	–
General psychopathology subscale score (16 items)	–	52.6 (11.6)	–	–
CPZ—chlorpromazine equivalent daily dosage	–	603.2 (397.6)	–	–
Disease duration (years)	–	9.9 (9.0)	–	–
Reaction time (ms)	423.76 (62.74)	614.43 (105.88)	107.85	<.001
Commission error (%)	9.8 (11.2)	32.2 (17.6)	51.64	<.001
Omission error (%)	1.1 (2.0)	20.4 (21.7)	38.89	<.001

a: Chi-square test for categorical, ANOVA for continuous variables.

b: 26 pairs of HC were examined, the EEG was not evaluable in 4 subjects due to artifacts.

c: 18 pairs of SZ were examined, the EEG was not evaluable in 2 subjects due to artifacts.

d: 1 = low, 2 = medium, 3 = high.

n/a: not applicable.

The mean PANSS total score in the patient group was 101.6 (SD = 20.7). The mean duration of illness was 9.9 years (SD = 9.0), and the mean chlorpromazine-equivalent antipsychotic dosage was 603.2 mg/day (SD = 397.6). At the time of the EEG recording, 64% of patients (n = 22) were receiving benzodiazepine treatment. PANSS subscale scores indicated comparable severity across symptom dimensions, with positive, negative, and general psychopathology scores of 23.7 (SD = 6.3), 25.3 (SD = 5.9), and 52.6 (SD = 11.6), respectively.

### 3.2 Electrophysiological results

#### 3.2.1 N400 analyses

##### 3.2.1.1 Group differences

**Figure 1:**
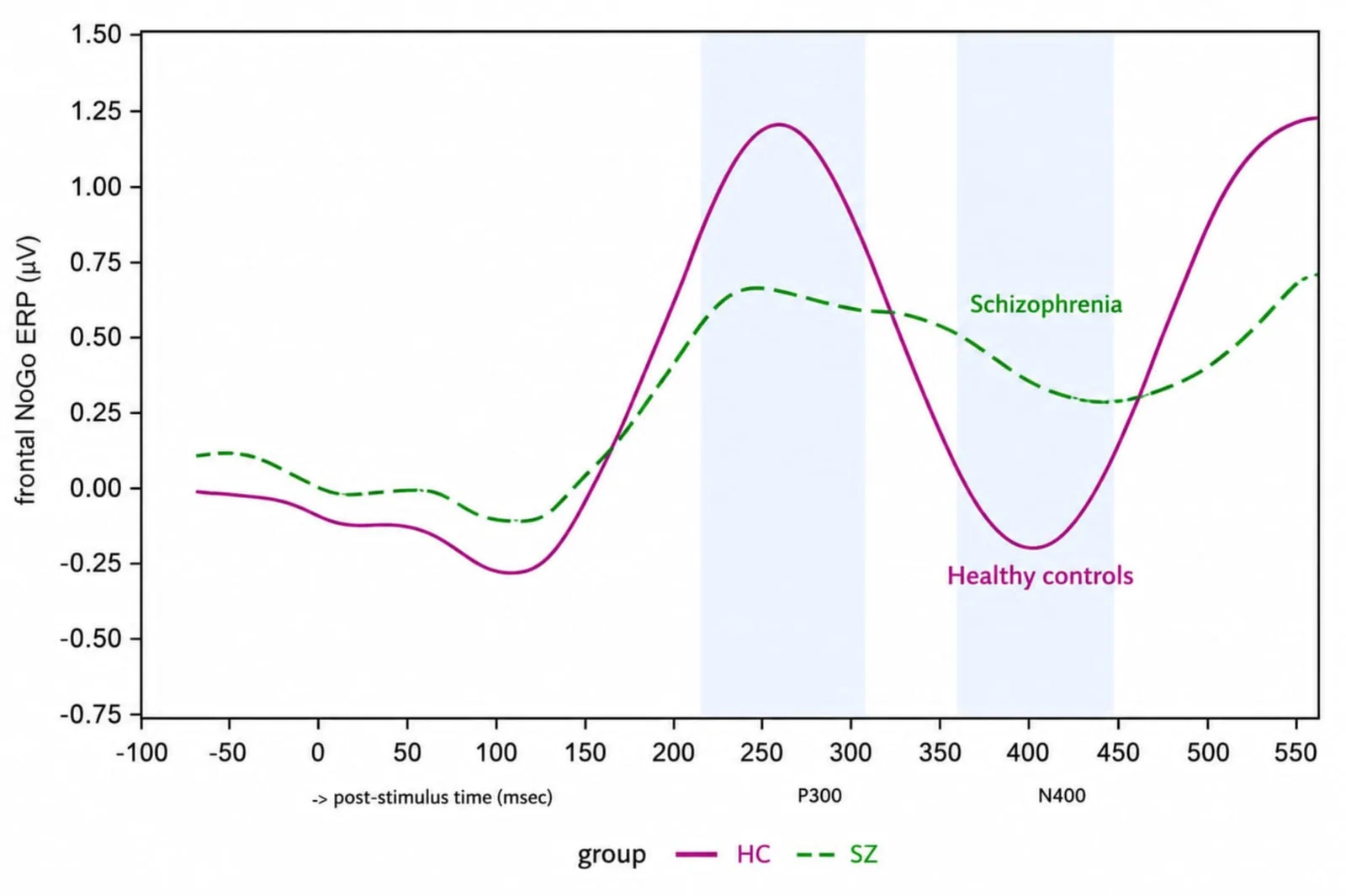
Fronto-central NoGo ERP in schizophrenia and healthy control groups across all stimuli. Fronto-central NoGo ERP waveforms for all stimuli combined in healthy controls and patients with schizophrenia during the combined analysis of the first and second task exposures: Patients with schizophrenia (green, dashed line) exhibited a significantly reduced amplitude in the N400 component compared to healthy controls (purple) and additionally showed a reduced P300 amplitude.

**Figure 2:**
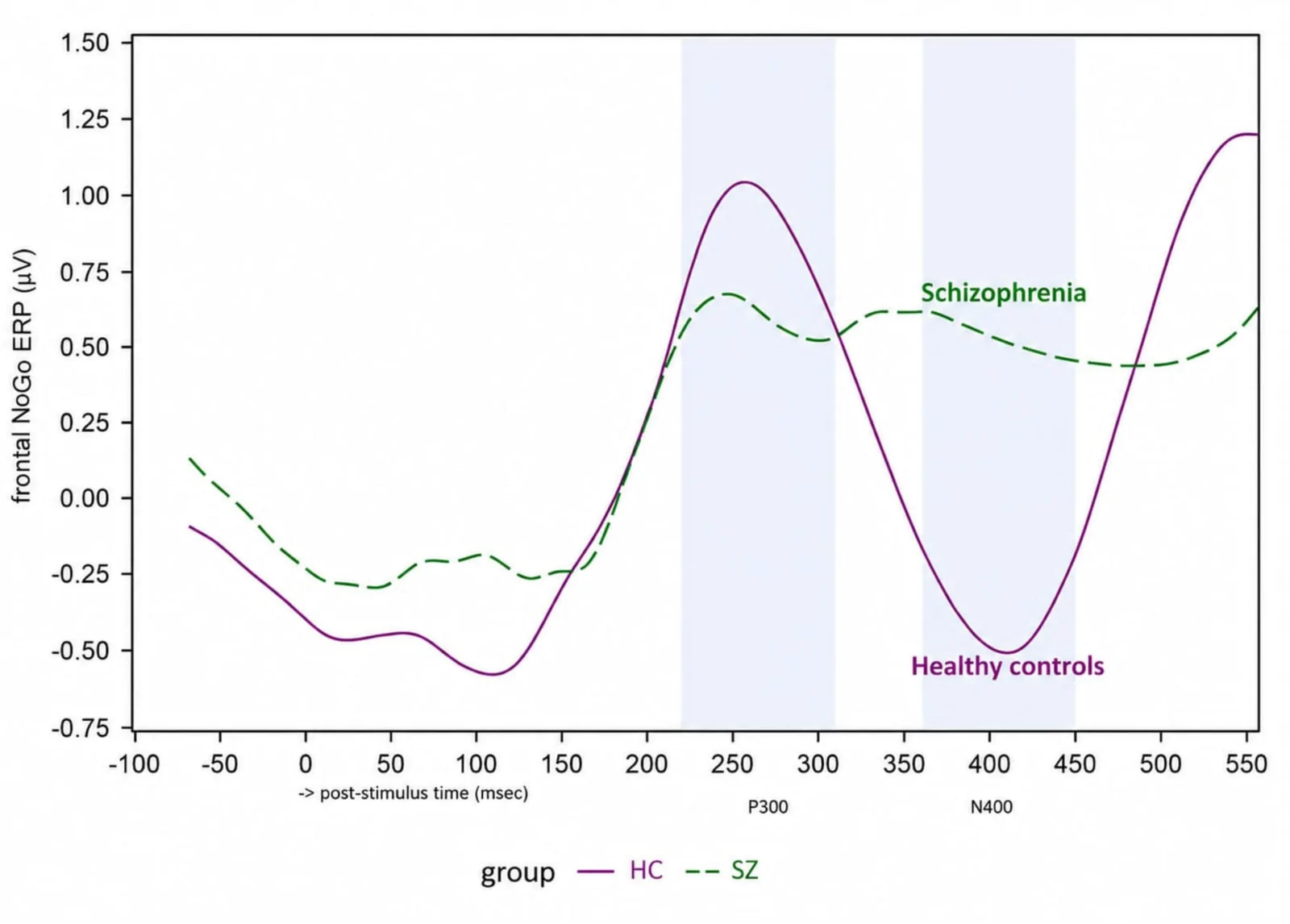
Fronto-central NoGo ERP in schizophrenia and healthy control groups for negative pictures. Fronto-central NoGo ERP waveforms for negative emotional stimuli in healthy controls and patients with schizophrenia during the combined analysis of the first and second task exposures: For negative stimuli, patients with schizophrenia (green, dashed line) exhibited a significantly reduced amplitude in the N400 component compared to healthy controls (purple), alongside a tendency toward a lower P300 amplitude.

**Figure 3:**
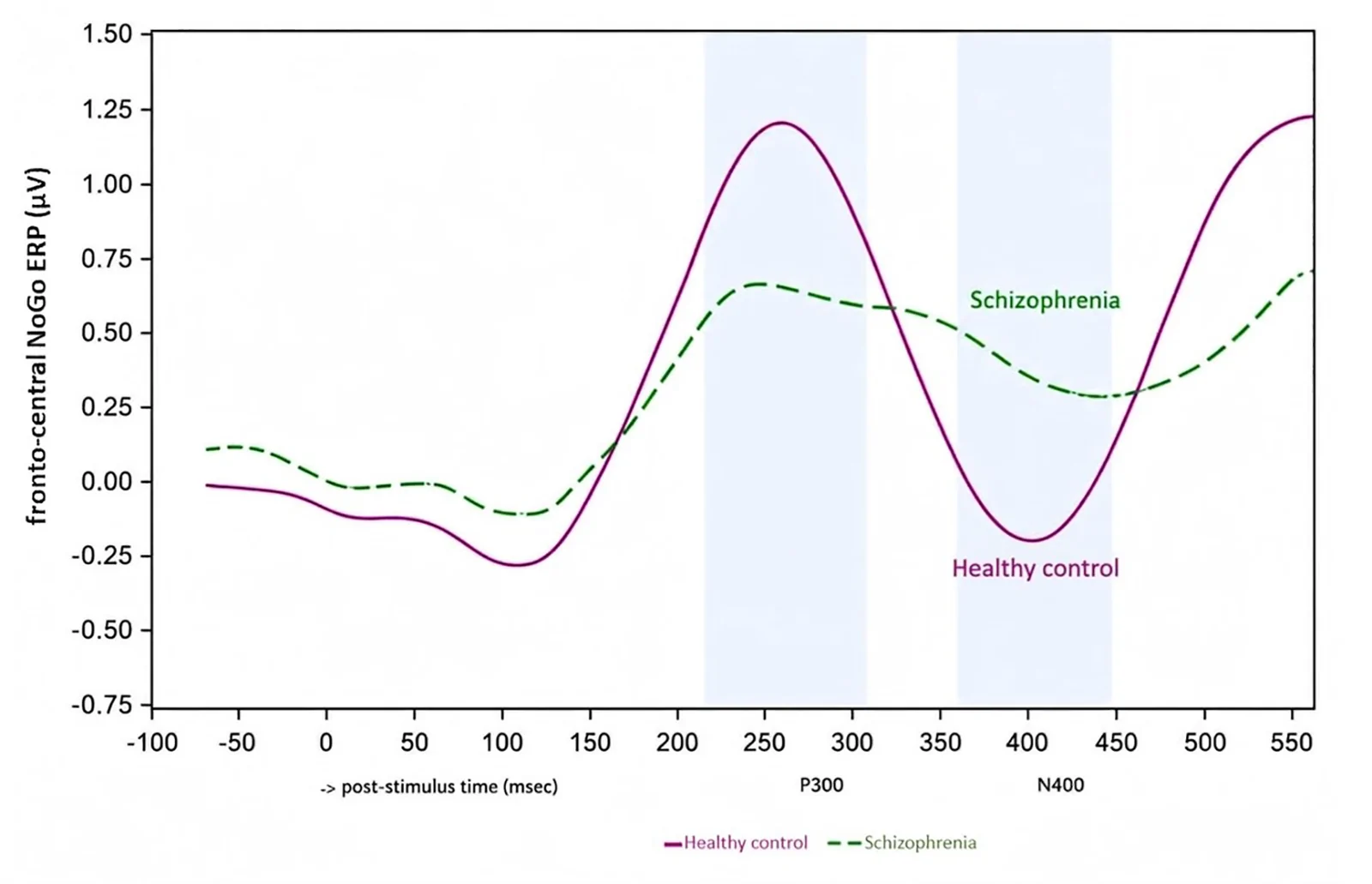
Fronto-central NoGo ERP in schizophrenia and healthy control groups for positive pictures. Fronto-central NoGo ERP waveforms for positive emotional stimuli in healthy controls and patients with schizophrenia during the combined analysis of the first and second task exposures: For positive stimuli, patients with schizophrenia (green, dashed line) exhibited a significantly reduced amplitude in the N400 component compared to healthy controls (purple), accompanied by a reduced P300 amplitude.

Regarding the N400 component, the combined analysis of the first and second exposures revealed a significant overall group difference across all stimuli combined (t = -1.97, p <.001, p_adj_ = .0004). Specifically, the SZ group exhibited a significantly reduced N400 amplitude compared to the HC group, with a mean difference of -0.6442 μV and a medium effect size (Cohen’s d = -0.442). For negative emotional stimuli, a significant overall group difference was also observed (t = -2.85, p <.001, p_adj_= .0004), with the SZ group exhibiting a significantly reduced N400 amplitude relative to the HC group (mean difference = -1.177 μV, Cohen’s d = -0.639). For positive emotional stimuli, a significant overall group difference emerged (t = -2.13, p = .006, p_adj_ = .018), with the SZ group showing a reduced N400 amplitude compared to the HC group (mean difference = -0.775 μV, Cohen’s d = -0.477). Conversely, no significant group differences were found for neutral stimuli (t = -0.72, p = .577, p_adj_ = .630; mean difference = -0.278 μV, Cohen’s d = -0.161). **Figures 1**–**3** illustrate the significant group differences observed for all stimuli combined, negative emotional stimuli, and positive emotional stimuli, respectively.

**Figure 4:**
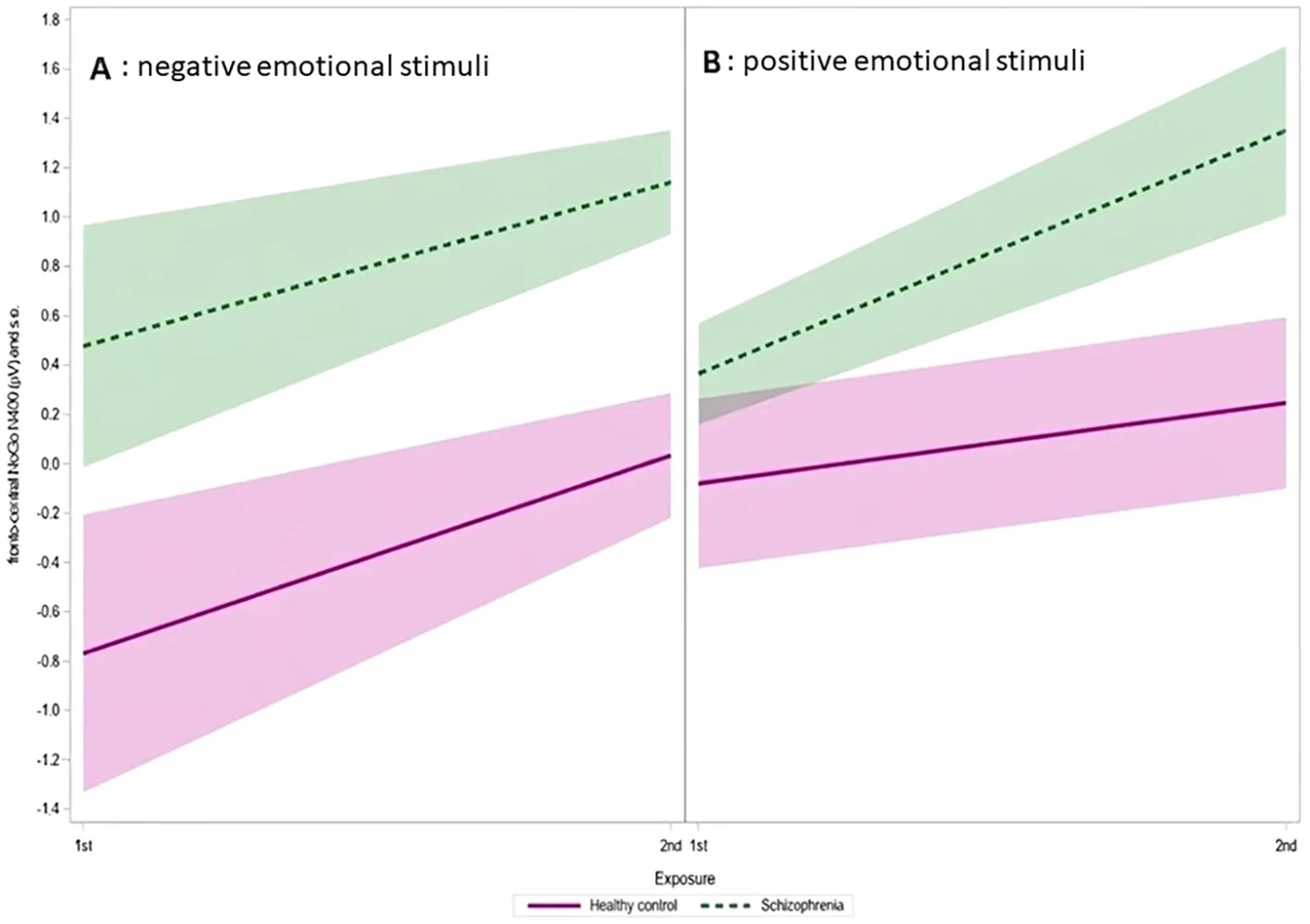
Fronto-central NoGo N400 amplitudes across first and second task exposures for negative emotional stimuli and positive emotional stimuli. Panels **(A)** and **(B)** display the grand average ERP waveforms for healthy controls and patients with schizophrenia elicited by negative emotional stimuli and positive emotional stimuli, respectively, during the first and second task exposures. The image shows the estimated means and standard errors (shaded areas) of the NoGo N400 component amplitude, derived from the HLM analysis. The difference between patients with schizophrenia (green, dashed line) and healthy controls (purple line) did not reach the level of statistical significance during the first task exposure for the negative emotional stimuli or positive emotional stimuli (*p* > 0.10). During the second task exposure, a statistically significant group difference emerged, as patients with schizophrenia exhibited a marked reversal of N400 polarity while the N400 amplitude did not significantly change across exposures in the healthy control group.

The temporal order of stimulus presentation modulated N400 amplitudes differently across the two groups. During the first exposure, the SZ group showed a numerically reduced N400 amplitude compared to the HC group, but this effect did not reach statistical significance across all stimuli combined (t = -1.00, p = .316, p_adj_ = .3794), nor for negative (t = -1.64, p = .100, p_adj_ = .1719) or positive emotional stimuli (t = -1.04, p = .299, p_adj_ = .3794). While N400 amplitude did not significantly change across exposures in the HC group, a statistically significant group difference emerged during the second exposure, characterized by a pronounced reversal of N400 polarity in the SZ group relative to the HC group, particularly for negative emotional stimuli, where a large effect size was observed (t = -4.42, p <.001, padj = .0004, Cohen’s d = -0.991), as well as for positive emotional stimuli, showing a medium effect size (t = -2.26, p = .024, padj = .0474, Cohen’s d = -0.507), and across all stimuli combined (t = -2.51, p = .012, padj = .0290, Cohen’s d = -0.563). The effect for neutral stimuli remained non-significant (t = -1.26, p = .207, p_adj_ = .3108). The group differences during the first and second exposure for the negative and positive emotional stimuli are shown respectively in panel A and B of **Figure 4**.

##### 3.2.1.2 Relationship with psychopathology

We examined the relationship between N400 amplitude and symptom severity as measured by the PANSS. In the combined analysis of the first and second exposures, the PANSS total score showed a significant negative association with fronto-central N400 amplitude (t = -2.19, p = .029, Cohen’s d = -0.39). Significant negative associations were also observed for PANSS negative symptoms (t = -2.57, p = .010, Cohen’s d = -0.45) and PANSS general symptoms (t = -2.08, p = .038, Cohen’s d = -0.36). In contrast, PANSS positive symptoms were not significantly associated with fronto-central N400 amplitude in the combined analysis (t = -1.16, p = .248, Cohen’s d = -0.21).

During the first exposure, no significant associations were observed between PANSS scores and N400 measures. During the second exposure, where significant group differences were observed, the PANSS total score showed a significant negative association with fronto-central N400 amplitude (t = -2.30, p = .0215, Cohen’s d = -0.41), indicating that higher overall symptom severity was associated with reduced N400. PANSS negative symptoms also showed a strong negative association with fronto-central N400 amplitude (t = -3.66, p = .0003, Cohen’s d = -0.65), with greater negative symptom severity linked to lower N400. Similarly, PANSS general symptoms were significantly negatively associated with fronto-central N400 amplitude (t = -2.20, p = .028, Cohen’s d = -0.38), whereas PANSS positive symptoms did not show a significant association (t = -1.10, p = .272, Cohen’s d = -0.19). Overall, higher symptom severity was consistently associated with a reduced N400 amplitude, whereas a polarity reversal relative to the HC group emerged at lower levels of psychopathology.

##### 3.2.1.3 Relationship with demographic and clinical characteristics

In terms of demographic variables, no significant effects of age (F(1,3648) = 0.32, p = .570) or gender (F(1,3648) = 0.22, p = .641) were observed on N400 amplitude. For the second exposure, duration of illness showed a significant negative association with N400 amplitude across all stimuli combined (F(1,672) = 6.08, p = .0139), whereas the association for negative emotional stimuli did not reach statistical significance (F(1,672) = 3.01, p = .0833).

With respect to medication, benzodiazepine use showed no association with N400 amplitude. However, when analyzing the second exposure, a significant negative association emerged between N400 amplitude and chlorpromazine-equivalent (CPZ) dose for all stimuli combined (F(1,672) = 5.47, p = .0196), indicating that higher antipsychotic doses were linked to smaller N400 responses. Although a similar trend was observed for negative emotional stimuli, this association did not reach statistical significance (F(1,672) = 2.76, p = .0971).

Importantly, CPZ-equivalent dose was not significantly correlated with duration of illness (r = .03, p = .89), suggesting that the association between CPZ-equivalent dose and N400 amplitude was not simply attributable to illness chronicity.

#### 3.2.2 P300 analyses

##### 3.2.2.1 Group differences

Consistent with the N400 findings, the temporal order of stimulus presentation differentially affected P300 amplitude in the SZ group and the HC group. Specifically, during the first exposure, P300 amplitude was significantly lower in the SZ group than in the HC group across all stimuli combined (t = 4.06, p <.001, Cohen’s d = 0.910) and for positive emotional stimuli (t = 2.77, p = .006, Cohen’s d = 0.621), while no significant difference emerged for negative stimuli (t = 0.46, p = .6476). However, during the second exposure, this group difference did not obtain significance for all stimuli combined (t = -0.17, p = .868) and positive stimuli (t = -0.27, p = .790), as P300 amplitude increased in the SZ group and decreased in the HC group. In contrast, a significant group difference emerged during the second exposure for negative emotional stimuli, showing a robust reduction in the SZ group (t = -2.69, p = .0072, Cohen’s d = -0.603).

## 4 Discussion

In this study, we employed EEG hyperscanning to investigate the neural underpinnings of social cognition within a social context. Specifically, we investigated the N400 event-related potential (ERP) elicited by emotional visual stimuli using an observer–actor paradigm. Unlike previous studies that have primarily examined N400 responses in isolated, single-person paradigms, our study evaluated N400 activity in the actor within a shared social context, focusing on the fronto-central electrode region of interest. By utilizing a hyperscanning setup—which enables simultaneous, temporally synchronized EEG recordings across individuals spanning from simple co-presence to complex interactions (24, 26, 46)—participants were measured within the same real-time social context. This social context can potentially enhance the ecological validity of the experimental situation, allowing us to better understand the neurobiological mechanisms underlying deficient information processing in a social setting (24, 25).

Regarding the behavioral results, patients with schizophrenia showed significantly prolonged reaction times as well as a significant increase in both omission and commission errors in the Go/NoGo task compared to healthy controls. These findings demonstrate that, consistent with findings from single-subject paradigms (47, 48), behavioral deficits in schizophrenia persist in simple social contexts.

Our primary result is that in patients with schizophrenia, a significant reduction in N400 amplitude was observed for all stimuli combined, as well as specifically for negative emotional stimuli with a large effect size and positive emotional stimuli with a medium effect size.

Our findings also indicate that the temporal order of stimulus presentation modulates the differences observed within this condition. Specifically, prior task performance exerted a significant, group-dependent effect on the subsequent N400 response. While the N400 remained stable during the second exposure in healthy controls, a pronounced reduction in N400 amplitude emerged in patients with schizophrenia. Furthermore, during the second exposure, the N400 amplitude showed a significant negative association with the PANSS total score, as well as with the severity of negative and general symptoms, and showed a similar numerical association with the severity of positive symptoms, though it did not reach the level of significance.

Specifically, higher symptom severity was associated with reduced (i.e., less negative) N400 amplitudes, consistent with impaired semantic integration and context-dependent processing. At lower levels of psychopathology, however, the N400 waveform exhibited a polarity reversal relative to healthy controls, suggesting that N400 abnormalities may involve qualitatively different pattern of neural response rather than a simple attenuation.

One possible explanation is the superposition of a negative-going N400 and a temporally overlapping P300 component. The observed ERP waveform can reflect a summation of these processes; thus, a relatively larger or more prolonged P300 may mask the N400, resulting in an apparent polarity reversal. This mechanism may be particularly relevant at lower symptom severity, where evaluative and attentional processes indexed by the P300 are relatively preserved or even exaggerated. In contrast, at higher levels of symptom severity, a more generalized disruption of cognitive processing may lead to attenuation of both components, yielding a reduced but not reversed N400.

Within this framework, the altered balance between N400 and P300 activity may reflect abnormalities in context updating and salience attribution. Such effects are consistent with models of aberrant salience and impaired sensory gating, which can increase processing demands and alter the allocation of attentional resources to incoming stimuli. Additionally, the negative association between N400 amplitude and disease duration during the second exposure is consistent with the view that schizophrenia involves progressive disruption of predictive, context-dependent semantic processing, suggesting that aberrant internal models of the environment may become increasingly entrenched over time.

A negative association was present between chlorpromazine-equivalent (CPZ) dose and N400 amplitude for all stimuli combined during the second exposure, with a similar, albeit non-significant, trend for negative emotional stimuli. Importantly, CPZ-equivalent dose was not significantly correlated with duration of illness, suggesting that the CPZ-N400 association was not simply attributable to illness chronicity. Nevertheless, this finding should be interpreted cautiously. Higher antipsychotic doses may reflect greater current clinical severity, negative or cognitive symptom burden, or medication-related psychomotor slowing, all of which could influence ERP responses. Therefore, the observed association cannot be taken as evidence of a direct suppressive effect of antipsychotic medication on the N400, but a direct or indirect medication-related contribution cannot be ruled out.

Emotional valence was also related to the magnitude of group differences, as N400 alterations in the schizophrenia group were most pronounced for negative stimuli, while still present for positive stimuli. This observation is consistent with evidence suggesting that emotionally valenced stimuli with negative and positive affective information are processed atypically in schizophrenia and may be especially vulnerable to distortions in salience attribution (20, 21, 49, 50). Together with the association between symptom severity and N400 amplitude, these results support the notion that aberrant salience contributes to disrupted contextual processing of emotionally negative information.

While the primary focus of the present study was the N400 response, secondary analyses of the P300 largely replicated previous findings. Patients with schizophrenia exhibited reduced P300 amplitudes during the first task exposure compared to healthy controls, consistent with earlier studies (51). During the second exposure, P300 amplitudes increased in patients but decreased in controls, replicating our previous observations (29). Emotional valence also appeared to influence the magnitude of group differences, with positive stimuli eliciting the most pronounced P300 alterations in the schizophrenia group during the first exposure and negative stimuli during the second exposure. This differential pattern may be consistent with altered habituation and salience allocation in schizophrenia, whereas the reduction in P300 amplitude observed in healthy controls is compatible with habituation across repeated presentations.

Regarding the enhancement of group differences during the second exposure, these dynamics point toward a potential failure of habituation rather than a simple task execution deficit. While healthy controls displayed expected physiological habituation, patients with schizophrenia exhibited an atypical pattern of N400 attenuation alongside an increase in P300 amplitude, aligning with our previous findings (29). Such an emergence of robust group differences during the second exposure suggests that the core effect may be less prominent in the initial processing of social-emotional information and may instead primarily affect the updating and maintenance of contextual representations over time. While healthy controls exhibited stable N400 responses across the second presentation, patients with schizophrenia showed attenuation and eventual polarity reversal of the N400. This pattern may reflect impaired adaptation to the social setting, resulting in aberrant salience attribution and increased recruitment of overlapping attentional-evaluative processes, as indexed by the concomitant increase in P300 amplitude.

Overall, our findings are consistent with data from the literature suggesting that the N400 component reflects the functioning of dynamic neural networks that are flexibly activated depending on context, and whose functioning is altered in schizophrenia spectrum disorders (52, 53). At the same time, our findings also highlight that in patients, not only the N400 but also the P300 component is altered, underlining that these two ERP components can be regarded as interdependent (30).

Nonetheless, it is important to note that the patients with schizophrenia participating in our study were in inpatient care and exhibited high levels of psychopathological symptoms at the time of assessment. In line with this clinical profile, they received relatively high-dose antipsychotic treatment, as indicated by the CPZ-equivalent dose. Although CPZ-equivalent dose was not significantly correlated with duration of illness, assessment during ongoing antipsychotic treatment limits our ability to disentangle the effects of medication, current symptom severity, negative or cognitive symptom burden, and psychomotor slowing on behavioral and electrophysiological measures. Therefore, medication-related effects should be considered when interpreting the observed behavioral and electrophysiological findings.

A further limitation of our study is that there was a difference in the male-to-female ratio between the HC and SZ groups. Although sex and educational level were included as covariates in the statistical models, residual confounding cannot be completely excluded. Consequently, replication in larger samples with more closely matched demographic characteristics is warranted, and the observed group differences should be interpreted with caution. Furthermore, the order of the actor and observer conditions should be considered when interpreting the exposure-related findings. As the second actor condition was preceded by exposure to the same stimuli during observation of another participant performing the task, the observed changes may reflect a broader exposure effect encompassing stimulus repetition, prior observation, and role order. The reduction in P300 amplitude in healthy participants may be consistent with habituation to repeated stimulus exposure, whereas the increase toward a larger response in patients suggests a different adaptation to repeated experience. The distinct patterns observed across ERP components and groups argue against a uniform order effect. Nevertheless, the individual contributions of stimulus repetition, prior observation, and role order cannot be disentangled in the present design.

Relatedly, the study did not include a single-subject condition in which the other participant was absent. Although our study did not involve direct interaction between participants, existing literature suggests that the mere presence of others can significantly modulate task performance (54, 55). Including a single-subject condition could help distinguish between the effects of mere social presence and active interaction; therefore, incorporating it would be an important direction for future research. Finally, our analysis focused on specific *a priori* selected electrode regions rather than full scalp topographic maps, which could provide additional spatial characterization of these ERP components in future studies.

Despite these limitations, our study offers new insights into N400 alterations in schizophrenia during social contexts, highlighting how these neurophysiological alterations are modulated by psychopathological severity and deficits in emotional processing. The observed N400 abnormalities are consistent with altered neural processes relevant to information processing and decision-making in a social context, rather than direct evidence of a primary social cognitive impairment. Although the exact relationship between N400 abnormalities and functional social cognitive deficits requires further investigation, particularly in longitudinal studies, our findings may contribute to a deeper understanding of the neurobiological mechanisms underlying social information processing and support the potential of EEG hyperscanning as a promising method for investigating these neural mechanisms in schizophrenia.

## Data Availability

The raw data supporting the conclusions of this article will be made available by the authors, without undue reservation.

## References

[1] Kahn RS Sommer IE Murray RM Meyer-Lindenberg A Weinberger DR Cannon TD . Schizophrenia. Nat Rev Dis Primers. (2015) 1:15067. doi: 10.1038/nrdp.2015.67 27189524

[2] Correll CU Schooler NR . Negative symptoms in schizophrenia: a review and clinical guide for recognition, assessment, and treatment. Neuropsychiatr Dis Treat. (2020) 16:519–34. doi: 10.2147/NDT.S225643 32110026 PMC7041437

[3] Green MF Horan WP Lee J . Social cognition in schizophrenia. Nat Rev Neurosci. (2015) 16:620–31. doi: 10.1038/nrn4005 26373471

[4] Green MF Bearden CE Cannon TD Fiske AP Hellemann GS Horan WP . Social cognition in schizophrenia, part 1: performance across phase of illness. Schizophr Bull. (2012) 38:854–64. doi: 10.1093/schbul/sbq171 21345917 PMC3406534

[5] Barbato M Liu L Penn DL Keefe RSE Perkins DO Woods SW . Social cognition as a mediator between neurocognition and functional outcome in individuals at clinical high risk for psychosis. Schizophr Res. (2013) 150:542–6. doi: 10.1016/j.schres.2013.08.015 24012459 PMC3839963

[6] Amoruso L Gelormini C Aboitiz F Alvarez González M Manes F Cardona JF . N400 ERPs for actions: building meaning in context. Front Hum Neurosci. (2013) 7:57. doi: 10.3389/fnhum.2013.00057 23459873 PMC3586681

[7] West WC Holcomb PJ . Event-related potentials during discourse-level semantic integration of complex pictures. Cognit Brain Res. (2002) 13:363–75. doi: 10.1016/S0926-6410(01)00129-X 11919001

[8] Kiang M Gerritsen CJ . The N400 event-related brain potential response: a window on deficits in predicting meaning in schizophrenia. Int J Psychophysiol. (2019) 145:65–9. doi: 10.1016/j.ijpsycho.2019.04.005 31047943

[9] Kutas M Federmeier KD . Thirty years and counting: finding meaning in the N400 component of the event-related brain potential (ERP). Annu Rev Psychol. (2011) 62:621–47. doi: 10.1146/annurev.psych.093008.131123 20809790 PMC4052444

[10] Jackson F Foti D Kotov R Perlman G Mathalon DH Proudfit GH . An incongruent reality: the N400 in relation to psychosis and recovery. Schizophr Res. (2014) 160:208–15. doi: 10.1016/j.schres.2014.09.039 25449716 PMC4258120

[11] Franklin MS Dien J Neely JH Huber E Waterson LD . Semantic priming modulates the N400, N300, and N400RP. Clin Neurophysiol. (2007) 118:1054–68. doi: 10.1016/j.clinph.2007.01.012 17336145

[12] Mohammad OM DeLisi LE . N400 in schizophrenia patients. Curr Opin Psychiatry. (2013) 26:196–207. doi: 10.1097/YCO.0b013e32835d9e56 23340116

[13] Kostova M Passerieux C Laurent JP Hardy-Baylé MC . N400 anomalies in schizophrenia are correlated with the severity of formal thought disorder. Schizophr Res. (2005) 78:285–91. doi: 10.1016/j.schres.2005.05.015 15993568

[14] Olichney JM Iragui VJ Kutas M Nowacki R Jeste DV . N400 abnormalities in late-life schizophrenia and related psychoses. Biol Psychiatry. (1997) 42:13–23. doi: 10.1016/S0006-3223(96)00242-9 9193737

[15] Jeon YW Polich J . Meta-analysis of P300 and schizophrenia: patients, paradigms, and practical implications. Psychophysiology. (2003) 40:684–701. doi: 10.1111/1469-8986.00070 14696723

[16] Bramon E Rabe-Hesketh S Sham P Murray RM Frangou S . Meta-analysis of the P300 and P50 waveforms in schizophrenia. Schizophr Res. (2004) 70:315–29. doi: 10.1016/j.schres.2004.01.004 15329307

[17] Kapur S . Psychosis as a state of aberrant salience: a framework linking biology, phenomenology, and pharmacology in schizophrenia. Am J Psychiatry. (2003) 160:13–23. doi: 10.1176/appi.ajp.160.1.13 12505794

[18] Murray GK Corlett PR Clark L Pessiglione M Blackwell AD Honey G . Substantia nigra/ventral tegmental reward prediction error disruption in psychosis. Mol Psychiatry. (2008) 13:239–76. doi: 10.1038/sj.mp.4002058 17684497 PMC2564111

[19] Almeida VN Radanovic M . Semantic priming and neurobiology in schizophrenia: a theoretical review. Neuropsychologia. (2021) 163:108058. doi: 10.1016/j.neuropsychologia.2021.108058 34655651

[20] Yildirim E Yalincetin B Sevilmis S Kutay O Alptekin K . Is there any relation between impaired emotion perception and thought disorder in schizophrenia? Noro Psikiyatr Ars. (2018) 55:118–22. doi: 10.5152/npa.2017.19277 30057451 PMC6060655

[21] Shateri L Yari Renani H Bakhshipour Rudsari A Hashemi Nosratabad T Saeidi Z . Through the lens of schizophrenia: recognizing negative facial expressions and family patterns. Schizophr Res Cognit. (2024) 39:100336. doi: 10.1016/j.scog.2024.100336 39559797 PMC11570855

[22] Sumiyoshi T Higuchi Y Itoh T Matsui M Arai H Suzuki M . Effect of perospirone on P300 electrophysiological activity and social cognition in schizophrenia: a three-dimensional analysis with sLORETA. Psychiatry Res. (2009) 172:180–3. doi: 10.1016/j.pscychresns.2008.07.005 19386475

[23] Riccardi C Montemagni C Del Favero E Bellino S Brasso C Rocca P . Pharmacological treatment for social cognition: current evidence. Int J Mol Sci. (2021) 22:7457. doi: 10.3390/ijms22147457 34299076 PMC8307511

[24] Ford JM Mathalon DH Kalba S Marsh L Pfefferbaum A . N1 and P300 abnormalities in patients with schizophrenia, epilepsy, and epilepsy with schizophrenia-like features. Biol Psychiatry. (2001) 49:848–60. doi: 10.1016/s0006-3223(00)01051-9 11343681

[25] Czeszumski A Eustergerling S Lang A Menrath D Gerstenberger M Schuberth S . Hyperscanning: a valid method to study neural inter-brain underpinnings of social interaction. Front Hum Neurosci. (2020) 14:39. doi: 10.3389/fnhum.2020.00039 32180710 PMC7059252

[26] Barde A Gumilar I Hayati AF Dey A Lee G Billinghurst M . A review of hyperscanning and its use in virtual environments. Informatics. (2020) 7:55. doi: 10.3390/informatics704005530654563

[27] Montague PR Berns GS Cohen JD McClure SM Pagnoni G Dhamala M . Hyperscanning: simultaneous fMRI during linked social interactions. Neuroimage. (2002) 16:1159–64. doi: 10.1006/nimg.2002.1150 12202103

[28] Hirata M Ikeda T Kikuchi M Kimura T Hiraishi H Yoshimura Y . Hyperscanning MEG for understanding mother-child cerebral interactions. Front Hum Neurosci. (2014) 8:118. doi: 10.3389/fnhum.2014.00118 24624076 PMC3941301

[29] Fullajtár M Kakuszi B Bitter I Czobor P . Alterations of NoGo P300 ERP in schizophrenia in social setting: a hyperscanning study. Transl Psychiatry. (2025) 15:270. doi: 10.1038/s41398-025-03481-6 40774954 PMC12331909

[30] Arbel Y Spencer KM Donchin E . The N400 and the P300 are not all that independent. Psychophysiology. (2011) 48:861–75. doi: 10.1111/j.1469-8986.2010.01151.x 21073481

[31] de la Asuncion J Bervoets C Morrens M Sabbe B De Bruijn ERA . EEG correlates of impaired self-other integration during joint-task performance in schizophrenia. Soc Cognit Affect Neurosci. (2015) 10:1365–72. doi: 10.1093/scan/nsv023 25759471 PMC4590534

[32] American Psychiatric Association DSM-5 Task Force . Diagnostic and Statistical Manual of Mental Disorders: DSM-5™. 5th ed. Washington, DC: American Psychiatric Publishing (2013).

[33] Derogatis LR Cleary PA . Factorial invariance across gender for the primary symptom dimensions of the SCL-90. Br J Soc Clin Psychol. (1977) 16:347–56. doi: 10.1111/j.2044-8260.1977.tb00241.x 588890

[34] Unoka Z Rózsa S Kő N Kállai J Fábián Á Simon L . Az SCL-90 tünetlista hazai standardizálása [Validity and reliability of the SCL-90 in a Hungarian population sample. Psychiatr Hung. (2004) 19:235–43.

[35] Langan J Martin D Shajahan P Smith DJ . Antipsychotic dose escalation as a trigger for neuroleptic Malignant syndrome: literature review and case series report. BMC Psychiatry. (2012) 12:214. doi: 10.1186/1471-244X-12-214 23194104 PMC3546951

[36] Kay SR Fiszbein A Opler LA . The positive and negative syndrome scale (PANSS) for schizophrenia. Schizophr Bull. (1987) 13:261–76. doi: 10.1093/schbul/13.2.261 3616518

[37] Marder SR Davis JM Chouinard G . The effects of risperidone on the five dimensions of schizophrenia derived by factor analysis: combined results of the North American trials. J Clin Psychiatry. (1997) 58:538–46. doi: 10.4088/jcp.v58n1205 9448657

[38] Hopkins SC Ogirala A Loebel A Koblan KS . Transformed PANSS factors intended to reduce pseudospecificity among symptom domains and enhance understanding of symptom change in antipsychotic-treated patients with schizophrenia. Schizophr Bull. (2018) 44:593–602. doi: 10.1093/schbul/sbx101 28981857 PMC5890480

[39] Lang PJ Bradley MM Cuthbert BN . International Affective Picture System (Iaps): Affective Ratings of Pictures and Instruction Manual. Gainesville, FL: University of Florida (2005). Technical Report A-6.

[40] Lang PJ Bradley MM Cuthbert BN . International Affective Picture System (Iaps): Affective Ratings of Pictures and Instruction Manual. Gainesville, FL: University of Florida (2008). Technical Report A-8.

[41] Barraza P Dumas G Liu H Blanco-Gomez G van den Heuvel MI Baart M . Implementing EEG hyperscanning setups. MethodsX. (2019) 6:428–36. doi: 10.1016/j.mex.2019.02.021 30906698 PMC6411510

[42] BioSemi B.V . Activetwo/Active2.5 System Specifications and Technical Documentation. Amsterdam, The Netherlands: BioSemi B.V. (2021). Available online at: https://www.biosemi.com/activetwo_full_specs.htm (Accessed April 7, 2026).

[43] Bryk AS Raudenbush SW . Hierarchical Linear Models: Applications and Data Analysis Methods. Newbury Park, CA: Sage Publications (1992).

[44] Gibbons RD Hedeker D Waternaux C Davis JM . Random regression models: a comprehensive approach to the analysis of longitudinal psychiatric data. Psychopharmacol Bull. (1988) 24:438–43. 3153505

[45] Benjamini Y Drai D Elmer G Kafkafi N Golani I . Controlling the false discovery rate in behavior genetics research. Behav Brain Res. (2001) 125:279–84. doi: 10.1016/s0166-4328(01)00297-2 11682119

[46] Dumas G . From inter-brain connectivity to inter-personal psychiatry. World Psychiatry. (2022) 21:214–5. doi: 10.1002/wps.20987 35524605 PMC9077612

[47] Green MF Horan WP Lee J . Nonsocial and social cognition in schizophrenia: current evidence and future directions. World Psychiatry. (2019) 18:146–61. doi: 10.1002/wps.20624 31059632 PMC6502429

[48] Lee SY Namkoong K Cho HH Song DH An SK . Reduced visual P300 amplitudes in individuals at ultra-high risk for psychosis and first-episode schizophrenia. Neurosci Lett. (2010) 486:156–60. doi: 10.1016/j.neulet.2010.09.035 20858531

[49] Roiser JP Stephan KE den Ouden HE Barnes TR Friston KJ Joyce EM . Do patients with schizophrenia exhibit aberrant salience? Psychol Med. (2009) 39:199–209. doi: 10.1017/S0033291708003863 18588739 PMC2635536

[50] Comparelli A Corigliano V Montalbani B Bargagna P Forcina F Cocco G . Relationship between aberrant salience and positive emotion misrecognition in acute relapse of schizophrenia. Asian J Psychiatr. (2020) 49:101975. doi: 10.1016/j.ajp.2020.101975 32114376

[51] Hamilton HK Mathalon DH Ford JM . P300 in schizophrenia: then and now. Biol Psychol. (2024) 187:108757. doi: 10.1016/j.biopsycho.2024.108757 38316196 PMC11686549

[52] Jacob MS Ford JM Roach BJ Calhoun VD Mathalon DH . Aberrant activity in conceptual networks underlies N400 deficits and unusual thoughts in schizophrenia. NeuroImage Clin. (2019) 24:101960. doi: 10.1016/j.nicl.2019.101960 31398555 PMC6699247

[53] Matsumoto Y Hayashi R Takahashi H . Understanding semantic impairments in schizophrenia from a predictive coding perspective. Neurosci Res. (2026) 222:104990. doi: 10.1016/j.neures.2025.104990 41270867

[54] Tian T Feng X Gu R Broster LS Feng C Wang L . Modulation of the brain activity in outcome evaluation by the presence of an audience: An electrophysiological investigation. Brain Res. (2015) 1615:139–47. doi: 10.1016/j.brainres.2015.04.040 25935695

[55] Luo Y Feng C Wu T Broster LS Cai H Gu R . Social comparison manifests in event-related potentials. Sci Rep. (2015) 5:12127. doi: 10.1038/srep12127 26183734 PMC4505307

